# Effect of serum electrolytes within normal ranges on QTc prolongation: a cross-sectional study in a Chinese rural general population

**DOI:** 10.1186/s12872-018-0906-1

**Published:** 2018-08-29

**Authors:** Yintao Chen, Xiaofan Guo, Guozhe Sun, Zhao Li, Liqiang Zheng, Yingxian Sun

**Affiliations:** 1 Department of Cardiology, The First Hospital of China Medical University, Shenyang, 110001 People’s Republic of China; 2 Department of Clinical Epidemiology, Library, Shengjing Hospital of China Medical University, Shenyang, Liaoning China

**Keywords:** QTc prolongation, Electrocardiography, Serum electrolytes, Serum potassium

## Abstract

**Background:**

Many previous clinical studies have reported that prolongation of the QT interval corrected for heart rate (QTc) is associated with an increased risk of sudden cardiac death and all-cause mortality. This study aimed to explore associations between serum electrolytes and QTc prolongation in the north-eastern Chinese rural general population.

**Methods:**

We performed a cross-sectional study including 10,334 (4820 men and 5514 women) from the general population aged ≥35 years in the Liaoning Province from 2012 to 2013. Anthropometric measurements, laboratory examinations and self-reported lifestyle factor information, echocardiography and electrocardiogram were collected by trained personnel. The associations between serum electrolytes and QTc prolongation were tested using multiple linear regression and logistic regression analyses.

**Results:**

The mean QTc interval were 415.6 ± 18.8 and 470.1 ± 23.1 ms in normal group and QTc prolongation group respectively. The prevalence of QTc prolongation increased significantly with a decrease in serum potassium and an increase in magnesium. Stepwise multiple linear regression showed that age, hypertension, waist circumference were prominently positive associated with QTc interval both in male and female population. But serum potassium was significantly inversely associated with QTc interval. Serum magnesium and calcium also showed a positive relationship with QTc interval. Furthermore, multiple logistic regression found that lower quartile of serum potassium had higher risk for QTc prolongation, especially in female population (Q2 vs. Q4: OR: 1.54, 95%CI: 1.01–2.35; Q1 vs. Q4: OR: 2.02, 95%CI: 1.36–3.01). In addition, the higher serum magnesium increased the risk of QTc prolongation, which was significantly only in male population.

**Conclusions:**

In present Chinese rural general population, even with normal range, a decrease in serum potassium and an increase in serum magnesium are important risk factors for QTc prolongation.

**Electronic supplementary material:**

The online version of this article (10.1186/s12872-018-0906-1) contains supplementary material, which is available to authorized users.

## Background

Many previous studies have concluded that prolongation of the QT interval corrected for heart rate (QTc) is associated with an increased risk of sudden cardiac death, arrhythmias and all-cause mortality [1–3]. At present, an electrocardiogram showing QTc prolongation is an important indicator for treatment and medication in clinical practice [4]. Many epidemiological risk factors for QTc prolongation have previously been reported, including older age, sex hormones and electrolyte disturbances [5–7]. Disturbances in serum electrolytes might induce or facilitate clinical arrhythmias by interacting with abnormal myocardial tissue, and this could even occur in a bundle of normal cardiac tissue. Furthermore, strong evidences has been found that identified hypokalaemia as a risk factor for QTc prolongation [8], and the dietary intake of potassium has also been independently associated with the QTc interval [9]. The same clinical studies also found that the concentrations of sodium, calcium and magnesium influence the QTc interval [7, 10, 11]. Therefore, electrolyte abnormalities might be an early sign of arrhythmia, including QTc prolongation. However, most of the relevant studies recruited participants from clinical patients, such as patients undergoing haemodialysis [10], patients with chronic kidney disease [12] or patients receiving psychotropic drugs treatment [13], who usually suffer from disturbances in serum electrolytes, and the number of participants was restricted to hundreds. Studies of the associations between serum electrolytes and QTc prolongation in a large sample from a Chinese general population have been rare. The characteristics of the general population and the above groups of patients are quite different which might result in very different conclusions, and the former population generally has normal levels of serum electrolytes. This present study aimed to explore associations between serum electrolytes and QTc prolongation in the north-eastern Chinese rural general population, which have not previously been reported to the best of our knowledge.

## Methods

### Study population

The detailed methods used in this study have been previously published [14]. A multi-stage, stratified randomly cluster-sampling scheme was adopted to build a representative sample of men and women in Liaoning Province that is located in Northeast China. From January 2012 to August 2013, we invited all the eligible permanent residents aged ≥35 years from each village to attend the study. Finally, 11,956 participants (85.3%) agreed and completed the present study. The study was approved by the Ethics Committee of China Medical University (Shenyang, China). All procedures were performed in accordance with the ethical standards. Written consent was obtained in all participants after they had been informed of the objectives, benefits, medical items and confidentiality agreement of personal information. If the participants were illiterate, we obtained the written informed consents from their proxies.

In present study, only participants with a complete set of data regarding the variables analyzed in the study were included, the exclusion criteria includes: recent use (two weeks before) of diuretics or diuretics-containing traditional Chinese medicine (*n* = 1056), recent use of unspecified antihypertensive drugs (*n* = 353), recent use of antipsychotics (*n* = 9), eGFR < 60 mL/min/1.73 m2 (*n* = 241), making a final sample size of 10,334 (4820 men and 5514 women).

### Data collection and measurements

The data collection and measurement methods used in this study have been previously described [14]. Data on demographic characteristics, lifestyle risk factors, dietary habits and any medicines used in previous 2 weeks were collected during a single visit by cardiologists and trained nurses using a standard questionnaire by a face-to-face interview. All eligible investigators must attend the organized training sessions in advance to maintain the importance of standardization and the study procedures. A strict test was administered after this training sessions, and only those who scored perfectly on the test could become investigators. During data collection, our inspectors received further instruction and support from a subcommittee for quality control of central steering committee.

The participants were advised to avoid caffeinated beverages and exercise for at least 30 min before the measurement. After a rest period of at least 5 min, blood pressure was measured three times at intervals of 2 min using a standardized automatic electronic sphygmomanometer (HEM-907; Omron, Japan). The mean of three measurements of blood pressure was calculated and used in all analyses. Hypertension was defined as a systolic blood pressure of 140 mmHg or greater, a diastolic blood pressure of 90 mmHg or greater, or self-reported current treatment for hypertension with antihypertensive medication [15].

Fasting blood samples were collected in the morning after at least 12 h of fasting for all participants. Blood samples were obtained from an antecubital vein into vacutainer tubes containing EDTA. Blood chemical analyses were performed at a central, certified laboratory. Serum electrolytes, fasting plasma glucose (FPG), lipid profiles, serum creatinine (SCr), and uric acid were analyzed enzymatically on an autoanalyzer (Olympus, Kobe, Japan). All laboratory equipment was calibrated and blinded duplicate samples were used. Diabetes mellitus was diagnosed according to the WHO criteria: FPG ≥ 7 mmol/L (126 mg/dL) and/or being on treatment for diabetes [16]. Dyslipidemia was defined according to the National Cholesterol Education ProgramThird Adult Treatment Panel (ATP III) criteria [17]. High total cholesterol (TC) was defined as TC ≥ 6.21 mmol/L (240 mg/dL). Low high density lipoprotein cholesterol (HDL-c) was defined as HDL-c < 1.03 mmol/L (40 mg/dL). High low density lipoprotein cholesterol (LDL-c) was defined as LDL-C ≥ 4.16 mmol/L (160 mg/dL). High triglyceride (TG) was defined as ≥2.26 mmol/L (200 mg/dL). The estimated glomerular filtration rate (eGFR) was estimated using the Chronic Kidney Diesease Epidemiology Collaboration (CKD-EPI) equation [18].

The method and standard of echocardiography measurements has been published by our previous article [19]. Left ventricular end-diastolic internal dimension (LVIDd), interventricular septal thickness (IVSd), posterior wall thickness (PWTd) were used to estimate the Left ventricular mass (LVM) by the Devereux’s formula according to the American Society of Echocardiography simplified cubed equation [20]. LVM = 0.8 × [1.04{(IVSTd +PWTd + LVIDd)^3^–LVIDd^3^}] + 0.6 g. Left ventricular mass index was obtained after that LVM was divided by height^2.7^. And left ventricular hypertrophy (LVH) was diagnosed using the following defining criteria [21]: > 48 g/m^2.7^ and 44 g/m^2.7^ for men and women respectively.

Twelve-lead resting, ten-second ECGs were performed on all participants by well-trained cardiologists using an ECG machine (MAC 5500; GE Healthcare, Little Chalfont, Buckinghamshire, UK). All ECGs were standard resting ECGs (25 mm/second paper speed and 10 mm/mV amplitude). After capturing images, QTc intervals were calculated and recorded automatically by the MUSE Cardiology Information System (version 7.0.0; GE Healthcare), the error of which determined by an automatic algorithm was less than ±20 ms. The accuracy was 99.98%, and the sensitivity was 99.62% [22]. In present study, Fridericia’s formula (QTc = QT/RR^1/3^) was used to correct the QT interval [23]. Prolonged QTc was defined according to the national guidelines, which recommended cut points of 450 milliseconds or longer in male and 460 milliseconds or longer in female [24].

### Statistical analysis

Continuous variables were expressed as mean values and standard deviation (SD), whereas categorical variables were described as frequencies and percentages. Comparisons between variables were analyzed by t-test or chi-square test as appropriate. The associations between serum electrolytes and prolonged QTc interval were tested using Pearson correlation, multiple linear regression and logistic regression analyses, with standard regression coefficient (β), odds ratio (OR) and 95% confidence intervals (CIs) calculated. All statistical analyses were performed using SPSS version 22.0 software (IBM Corp., Armonk, NY, USA), and *P* < 0.05 indicated statistical significance.

## Results

The population characteristics by QTc prolongation were showed in Table 1 and Additional file 1: Table S1. The prevalence of QTc prolongation in total participants was 4.1%. Participants with QTc prolongation were more likely to be older, higher WC, FPG, TC, HDL-C, LDL-C, blood pressure (SBP and DBP), LVMI_ht2.7_, serum calcium and serum magnesium, but lower eGFR and mean serum potassium (*P* < 0.05). The mean QTc interval were 415.6 ± 18.8 and 470.1 ± 23.1 ms in normal group and QTc prolongation group respectively.

**Table 1:** Characteristics in population with or without QTc prolongation

Variables	Normal QTc	QTc prolongation	*P* value
n (%)	9913 (95.9)	421 (4.1)	
Age, years	52.9 ± 10.2	58.6 ± 11.0	< 0.001
Gender(%)			
Male	4631 (46.7)	189 (44.9)	0.463
Female	5282 (53.3)	232 (55.1)	
Race (Han) (%)	9390 (94.7)	393 (93.3)	0.219
Current smoking (%)	3546 (35.8)	150 (35.6)	0.953
Current drinking (%)	2303 (23.2)	110 (26.1)	0.169
Physical activity (%)			
Low	2766 (27.9)	134 (31.8)	0.212
Moderate	6590 (66.5)	264 (62.7)	
Heavy	557 (5.6)	23 (5.5)	
Body mass index (kg/m2)	24.6 ± 3.6	24.9 ± 3.5	0.132
waist circumference (cm)	81.9 ± 9.7	83.8 ± 9.5	< 0.001
Diet score	2.3 ± 1.1	2.3 ± 1.1	0.451
LDL-cholesterol, mmol/L	2.9 ± 0.8	3.0 ± 0.8	0.004
HDL-cholesterol, mmol/L	1.4 ± 0.4	1.5 ± 0.4	0.002
Triglycerides, mmol/L	1.6 ± 1.5	1.6 ± 1.3	0.741
Total cholesterol (mmol/L)	5.2 ± 1.1	5.3 ± 1.1	0.017
Fasting glucose, mmol/L	5.8 ± 1.6	6.1 ± 2.2	0.06
Systolic blood pressure, mmHg	139.2 ± 22.0	152.8 ± 27.6	< 0.001
Diastolic blood pressure, mmHg	81.2 ± 11.3	83.9 ± 13.8	< 0.001
Estimated GFR (mL/min/1.73m^2^)	94.3 ± 14.3	92.0 ± 13.9	0.001
Serum uric acid, mmol/L	287.4 ± 82.0	293.0 ± 84.5	0.176
Serum sodium, mmol/L	141.2 ± 2.2	141.4 ± 2.4	0.124
Serum potassium, mmol/L	4.2 ± 0.3	4.1 ± 0.4	< 0.001
Serum calcium, mmol/L	2.32 ± 0.1	2.34 ± 0.1	< 0.001
Serum magnesium, mmol/L	0.8 ± 0.1	0.9 ± 0.1	0.022
LVMI_ht2.7_, g/m^2.7a^	39.2 ± 28.3	43.1 ± 12.5	0.005
QTc Fredericia, ms	415.6 ± 18.8	470.1 ± 23.1	< 0.001
QRS duration> 120 ms	117 (1.2)	67 (15.9)	< 0.001

Values are mean (SD) unless otherwise indicated. *P*-values represent the result of standard T test or Pearson chi-square test to detect differences between the groups. The echocardiography measurements were showed in Additional file 1: Table S1

**Fig. 1:**
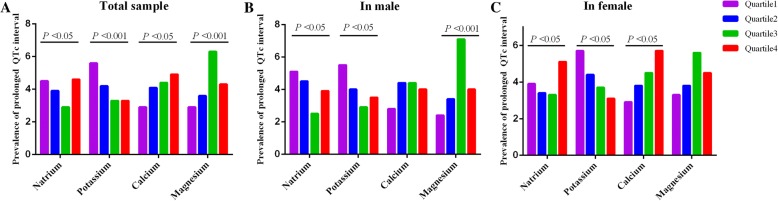
The prevalence of prolonged QTc interval in quartiles of serum electrolytes. Data is presented in total sample (**A**), male (**B**) and female (**C**) participants.The serum electrolytes,natrium, potassium, calcium and magnesium, are grouped by quartile. *P* value indicated the prevalence difference among the quartiles of each serum electrolytes. The thresholds for each quartile of serum electrolytes are showed in Table 3

Considered the gender difference for QTc prolongation, the different prevalence in male and female grouped by quartiles of electrolytes was analyzed (Fig. 1). Both in male and female, the prevalence of QTc prolongation was increasing significantly with a decrease in the concentration of serum potassium, which was opposite to serum total calcium in female and magnesium in total sample. And the prevalence of QTc prolongation was high to 5.7% in female population with lowest quartile of serum potassium. It seemed that both lower and higher serum sodium would increase the prevalence of QTc prolongation.

Stepwise multiple linear regression was used to analysis the risk factors of QTc prolongation (Table 2). Lots of potential risk factors were included in the regression model, and it was showed that age, hypertension, waist circumference were prominently positive associated with QTc interval in both male and female population. But serum potassium was significantly inversely associated with QTc interval in both male and female. Serum magnesium and calcium also showed a positive relationship with QTc interval. But diabetes and serum uric acid was more positive relevant for QTc prolongation just in male. And serum sodium did not show significant association with QTc interval, which was removed from the regression model finally.

**Table 2:** Stepwise multiple linear regression for associations between coronary risk factors and QTc interval

	Male			Female		
Variables	Coef^a^	SE^b^	beta^c^	Coef^a^	SE^b^	beta^c^
Age (per 10 years)	4.98	0.35	0.25	1.82	0.38	0.08
current smoking (yes/no)	1.55	0.60	0.04	3.60	0.80	0.06
waist circumference	0.20	0.03	0.09	0.23	0.03	0.10
Hypertension	5.58	0.63	0.13	3.34	0.64	0.08
Diabetes (yes/no)	2.66	1.04	0.04	–	–	–
Estimated GFR	0.09	0.03	0.06	0.09	0.03	0.06
Serum uric acid	0.01	0.004	0.05	–	–	–
Serum calcium	5.77	2.44	0.04	10.72	2.34	0.07
Serum potassium	−7.76	0.85	−0.13	−6.44	0.88	−0.10
Serum magnesium	20.23	3.87	0.08	9.93	2.28	0.06
LVMI_ht2.7_	–	–	–	0.03	0.01	0.04

The short dashes meaned that the variable was removed by the stepwise process, and there were other adjusted variables including dietscore, current drinking, BMI, physical activity, dyslipidaemia, and serum sodium which also removed from all the models finally. ^a^ unstandardized coefficient, ^b^standard error, ^c^standardized beta. , and the *P* <0.05 for all β values in the table

Furthermore, multiple logistic regression (Table 3) was used to explore the relationships between serum electrolytes and QTc prolongation. It was found that lower quartile of serum potassium had higher risk for QTc prolongation, especially in female population (Q2 vs. Q4: OR: 1.54, 95%CI: 1.01–2.35; Q1 vs. Q4: OR: 2.02, 95%CI: 1.36–3.01). In addition, the higher serum magnesium also increased the risk of prolonged QTc in total population and male population. Compared with the lowest quartile of sodium, only the quartile 3 showed negative association with QTc prolongation in male participants (OR: 0.47, 95%CI: 0.28–0.80). But calcium didn’t show any statistically positive association with QTc prolongation.

**Table 3:** Multiple logistic regression for associations between quartiles of serum electrolytes and QTc prolongation

		Total sample			Male			Female		
	Thresholds (mmol/L)	OR	Lower	Upper	OR	Lower	Upper	OR	Lower	Upper
Serum sodium										
Quartile1	<140.0	Reference			Reference			Reference		
Quartile2	140.0-140.9	0.89	0.63	1.24	0.84	0.53	1.34	1.00	0.61	1.65
Quartile3	141.0-141.9	*0.61* ^*^	*0.43* ^*^	*0.87* ^*^	*0.47* ^*^	*0.28* ^*^	*0.80* ^*^	0.83	0.50	1.37
Quartile4	> 142.0	0.83	0.62	1.11	0.67	0.44	1.03	1.11	0.72	1.72
Serum potassium										
Quartile1	<4.00	*1.72* ^*^	*1.30* ^*^	*2.28* ^*^	*1.67* ^*^	*1.10* ^*^	*2.54* ^*^	*2.02* ^*^	*1.36* ^*^	*3.01* ^*^
Quartile2	4.00-4.19	1.32	0.98	1.78	1.19	0.77	1.85	*1.54* ^*^	*1.01*	*2.35*
Quartile3	4.20-4.39	1.04	0.76	1.43	0.85	0.53	1.37	1.27	0.83	1.95
Quartile4	> 4.40	Reference			Reference			Reference		
Serum Calcium										
Quartile1	<2.24	Reference			Reference			Reference		
Quartile2	2.24-2.32	1.30	0.94	1.79	1.60	0.99	2.60	1.09	0.70	1.70
Quartile3	2.33-2.41	1.28	0.92	1.78	1.41	0.85	2.34	1.19	0.76	1.86
Quartile4	> 2.42	1.30	0.92	1.83	1.16	0.68	1.99	1.40	0.89	2.22
Serum magnesium										
Quartile1	<0.80	Reference			Reference			Reference		
Quartile2	0.80-0.83	1.33	0.91	1.95	1.83	0.97	3.46	1.13	0.70	1.83
Quartile3	0.84-0.89	*1.88* ^*^	*1.20* ^*^	*2.93* ^*^	*4.06* ^*^	*2.00* ^*^	*8.22* ^*^	1.05	0.58	1.91
Quartile4	> 0.90	*1.55* ^*^	*1.06* ^*^	*2.26* ^*^	*2.47* ^*^	*1.32* ^*^	*4.64* ^*^	1.21	0.74	1.96

All the serum electrolytes were adjusted by each other in the same model. The other adjustment factors included age, serum uric acid, body mass index, waist circumference, race, diet score, current smoking, current drinking, physical activity, eGFR, hypertension, dyslipidaemia, diabetes and LVH

*OR* odds ratio

^*^P < 0.05

## Discussion

It has been observed that electrolyte disturbances may trigger or facilitate clinical arrhythmias that might result in sudden death, even in a bundle of normal cardiac tissue [25]. Previous clinical studies found that changes in the QTc interval were inversely associated with levels of serum potassium and magnesium after dialysis [13] and a low Ca^2+^ concentration in the dialysate [10]. QTc prolongation has also been considered to increase the risk of ventricular arrhythmia and sudden death in clinical patients affected by acquired or genetic long QT syndrome [10], which is also presented in the healthy population [26]. However, studies that reported the association between serum electrolytes and the QTc prolongation in a general population have been rare. In the present general population, in contrast to previous patient groups, stepwise linear regression showed that QTc prolongation had a positive relationship with serum magnesium and calcium, and a prominent negative relationship with of serum potassium in both male and female sbjects. However, after adjustment for other factors, multiple logistic regression only found a significant inverse association between serum potassium and QTc prolongation in both male and female subjects, and a positive association between serum magnesium and QTc prolongation in male subjects alone. Regarding the general population in the present study, the first quartile cut-off for serum potassium (4 mmol/L) was much higher than the clinical diagnostic threshold for hypokalaemia (3.5 mmol/L), and the prevalence of hypokalaemia in this population was only 0.9% (*n* = 95). The prevalences of hyperkalaemia (0.2%, *n* = 21, > 5.3 mmol/L) and hypermagnesemia (0.1%, *n* = 6, > 1.28 mmol/L) were even lower. Hence, the vast majority of the participants who were examined had normal levels of serum potassium and magnesium. However, we also found that even when the ranges were normal, a decrease in serum potassium and an increase in serum magnesium increased the risk of QTc prolongation in the present general population.

It is well known that the QTc interval is regulated or affected by dysfunction of the cardiac autonomic nervous system [27]. The sympathetic and parasympathetic nervous systems control the force of heart contractions by influencing calcium channels and control the heart rate via potassium channels, as well as controlling atrioventricular transduction [28]. It has previously been reported that two-pore-domain-potassium (K2P) channels are also expressed in the autonomic nervous system, where they might be important modulators of neuronal excitability [29]. In addition, as the most abundant intracellular cation, potassium is the key determinant of the resting membrane potential. The potassium gradient and the concentration of other electrolytes could modulate potassium currents, which might result in abnormal electrocardiographic findings [25]. Thus, serum potassium could influence the QTc interval directly by modulating potassium currents or indirectly by affecting cardiac the autonomic nervous system.

One important characteristic of this general population is that nearly half of the population suffered from hypertension. They also lived in north-eastern China, which is an area with a high dietary sodium intake. It has previously been reported that individuals with hypertension displayed QTc prolongation or QTc dispersion [30]. Some researchers have postulated that sympathetic predominance and chronic anxiety could increase QTc prolongation in a hypertensive population. In accordance with previous studies, we also found a significantly positive association between hypertension and QTc prolongation. Because of the relatively poor economic conditions of the present rural population, these hypertensive patients usually selected diuretics or traditional Chinese medicines (containing diuretics) for treatment, which might result in electrolyte disturbance including low serum potassium, and thus increase the risk of QTc prolongation. In particular for the male population with hypertension, a decrease serum potassium within the normal range was also a risk indicator. More attention should be paid during antihypertensive therapy.

In clinical studies, the risk of QTc prolongation could be decreased by potassium supplementation in patients with hypokalaemia, or by an appropriate increase in the concentration of potassium in the dialysate in patients undergoing heamodialysis [10]. However, in the general population, the vast majority of participants had normal levels of serum electrolytes. It would be inappropriate to suggest that they take potassium supplements to reduce the risk of QTc prolongation, which might lead to another risk status, namely, hyperkalaemia. Previous studies found that a low dietary potassium intake was independently associated with QTc prolongation [9], and a significantly higher incidence of cardiovascular disease and mortality [31]. Hence, it might be advisable to consume foods rich in potassium to reduce the risk of QTc prolongation.

A previous study reported that the duration of the post-dialysis QTc interval was inversely correlated with the change in magnesium levels after dialysis [11], which was established on the basis of single-factor analysis in 50 patients with end-stage renal disease who were undergoing regular hemodialysis. However, another study reported that lower levels of serum magnesium were related to a less pronounced increase in the QTc interval in patients with aneurysmal subarachnoid haemorrhage [32]. However, in this study, we found a positive relationship between serum magnesium and QTc prolongation in the general population without eGFR < 60 mL/min/1.73m^2^. The clinical observational studies had limited sample sizes and more selection bias, which might be the causes of the difference in the results. The results of our study indicated that an increase of serum magnesium within the normal range might also increase the risk of QTc prolongation in the male population.

The major limitation of the study was a cross-sectional analysis which only made assessment of associations possible and couldn’t determine the causal effect. Second, the ECG examination was taken at one occasion only. The confounding effects of systematic error in measurement and morphology of QT intervals could not be ruled out. And due to large population, in this study the analysis has been done from computer measured QTc interval which in several studies have shown differences with manually measured QTc. Third, serum electrolytes were measured only once that might be affected by other confounding factors including dietary intake of electrolytes and urinary electrolyte excretion. But this population was a large sample general population which decreased the selection bias and provided more informative reference values.

## Conclusions

In conclusions, the prevalence of QTc prolongation is significantly higher in participants with lower serum potassium and higher magnesium. In present Chinese rural general population, even with normal range, a decrease in serum potassium and an increase in serum magnesium are important risk factors for QTc prolongation. Thus, causes of reducing serum potassium should be paid more attention, especially for female individuals. And the positive association between serum magnesium and QTc prolongation in male population needs more studies to elucidate.

## Additional file

Additional file 1: **Table S1.** The echocardiography data with or without QTc prolongation. (DOCX 16 kb)

## Abbreviations

BMI: Body mass index
ECGs: Electrocardiograph
eGFR: Estimated glomerular filtration rate
FPG: Fasting plasma glucose
HDL-c: High density lipoprotein cholesterol
LDL-c: Low density lipoprotein cholesterol
LVH: Left ventricular hypertrophy
LVM: Left ventricular mass
QTc: Corrected-QT interval
SCr: Serum creatinine
TC: Total cholesterol
TG: Triglyceride
WC: Waist circumference
