# Supplementary material for: Effect of serum electrolytes within normal ranges on QTc prolongation: a cross-sectional study in a Chinese rural general population
Source: BMC Cardiovasc Disord. 2018 Aug 29;18:175. doi: 10.1186/s12872-018-0906-1 (PMC6114040; doi:10.1186/s12872-018-0906-1)
Supplement: Supplementary file 1 — Table S1. The echocardiography data with or without QTc prolongation. (DOCX 16 kb) [file 12872_2018_906_MOESM1_ESM.docx]

| Table S1. The echocardiography data with and without QTc prolongation | | | |
| --- | --- | --- | --- |
| Variables | Normal QTc | Prolonged QTc | P value |
| n (%) | 9913 (95.9) | 421 (4.1) |  |
| End-diastolic left ventricular internal dimension (LVIDd), mm | 4.7 ± 0.5 | 4.8 ± 0.5 | <0.001 |
| End-diastolic interventricular septum thickness (IVSTd), mm | 0.9 ± 0.3 | 0.9 ± 0.1 | 0.005 |
| End-diastolic posterior wall thickness (PWTd), mm | 0.9 ± 0.3 | 0.9 ± 0.3 | 0.004 |
| Left ventricular mass (LVM) | 141.0 ± 102.4 | 152.2 ± 45.8 | 0.027 |
| Values are mean (SD) unless otherwise indicated. P-values represent the result of standard T test or Pearson chi-square test to detect differences between the groups. | | | |
